# Death of Dr. John McCurdy

**Published:** 1867-06

**Authors:** 


### DEATH OF DR. JOHN R. McCURDY.

The heading of this is not penned as a news item. The death of John R. McCurdy is doubtless known to nearly all our readers. He died on the 27th of last January, of consumption, at his home in Philadelphia.

When the sad event occurred, the writer of this had but a dim prospect of life; and the news, so painful to others, suggested to him that a friend had gone before. The career of Dr. McCurdy was a highly successful one, and was so strongly enstamped on the Dental profession that its impressions will be ever recognized. As a member of the firm of Jones, White & McCurdy, as editor of the "Dental News Letter," as a friend to the profession, an active co-operator in all that tended to elevate and advance its interests, he had few equals. His was an active, earnest, energetic life. The world is the better for his having lived. W.

